# Before Translating Extracellular Vesicles into Personalized Diagnostics and Therapeutics: What We Could Do

**DOI:** 10.1021/acs.molpharmaceut.4c00185

**Published:** 2024-05-21

**Authors:** Chi-An Cheng

**Affiliations:** † School of Pharmacy, College of Medicine, National Taiwan University, Taipei 10050, Taiwan

**Keywords:** extracellular vesicle, exosome, protein biomarker, diagnostics, drug delivery, personalized medicine, single molecule array

## Abstract

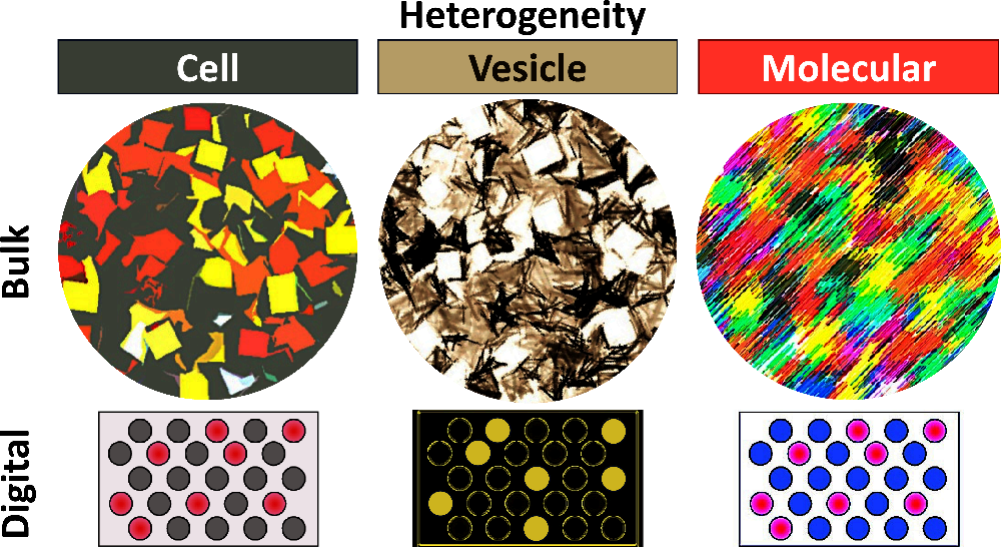

Extracellular vesicle (EV) research is rapidly advancing from fundamental science to translational applications in EV-based personalized therapeutics and diagnostics. Yet, fundamental questions persist regarding EV biology and mechanisms, particularly concerning the heterogeneous interactions between EVs and cells. While we have made strides in understanding virus delivery and intracellular vesicle transport, our comprehension of EV trafficking remains limited. EVs are believed to mediate intercellular communication through cargo transfer, but uncertainties persist regarding the occurrence and quantification of EV-cargo delivery within acceptor cells. This ambiguity is crucial to address, given the significant translational impact of EVs on therapeutics and diagnostics. This perspective article does not seek to provide exhaustive recommendations and guidance on EV-related studies, as these are well-articulated in position papers and statements by the International Society for Extracellular Vesicles (ISEV), including the ‘Minimum Information for Studies of Extracellular Vesicles’ (MISEV) 2014, MISEV2018, and the recent MISEV2023. Instead, recognizing the multilayered heterogeneity of EVs as both a challenge and an opportunity, this perspective emphasizes novel approaches to facilitate our understanding of diverse EV biology, address uncertainties, and leverage this knowledge to advance EV-based personalized diagnostics and therapeutics. Specifically, this perspective synthesizes current insights, identifies opportunities, and highlights exciting technological advancements in ultrasensitive single EV or “digital” profiling developed within the author’s multidisciplinary group. These newly developed technologies address technical gaps in dissecting the molecular contents of EV subsets, contributing to the evolution of EVs as next-generation liquid biopsies for diagnostics and providing better quality control for EV-based therapeutics.

## 1 Introduction

**Figure 1:**
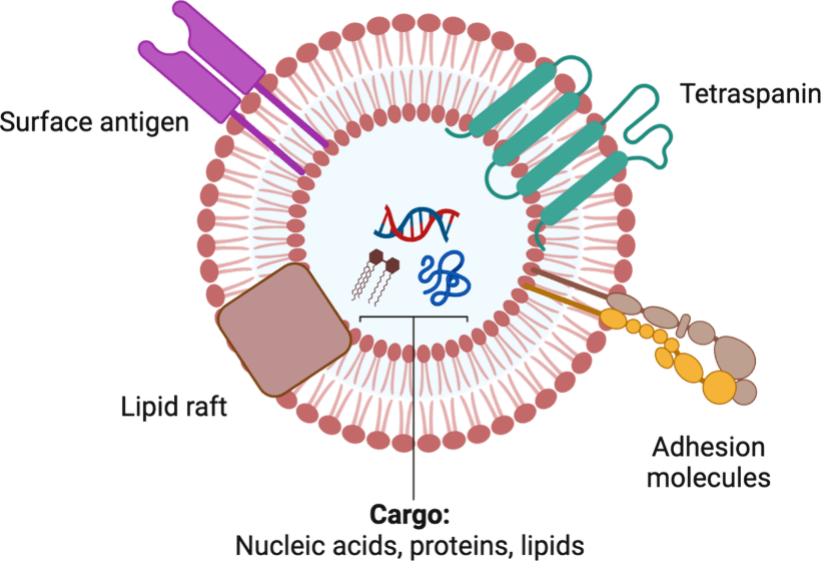
**Typical structure and biomolecular cargo of an extracellular vesicle (EV).** This vesicle carries a diverse payload, which can consist of various nucleic acids (such as miRNAs, mRNAs, and DNAs), proteins, lipids, receptors, adhesion molecules, and other biomolecules.

Extracellular vesicles (EVs) are nanosized particles enclosed by lipid bilayers released into circulation by all cell types, including tumor cells.^1^ These vesicles carry a diverse cargo, including signal proteins, receptors, effector proteins, DNA, RNA, and lipids (Figure 1). In addition to the conventional secretory pathway, EVs play a crucial role in intercellular signaling, acting as vehicles for transmitting information to various targets.^2^ The term “EVs” is recommended by the International Society for Extracellular Vesicles (ISEV), encompassing a heterogeneous population of vesicles. Terms like exosomes or ectosomes should only be employed when their biogenesis has been thoroughly established.^1^ Unlike classical intercellular communication, where cells secrete specific molecules as signals, EVs deliver complex signals and information to acceptor cells, eliciting multifaceted cellular responses.^2,3^

EVs serve as “packages” of information, capable of transporting their cargo over long distances.^4−6^ Initially, it was believed that candidates for genetic information transfer were those capable of amplification, such as mRNAs. These molecules can be translated repeatedly to produce a multitude of proteins, thus exerting an effect even if only a few mRNA molecules enter the cell.^4,6,7^ Many groups also confirmed the presence of miRNAs in EVs and demonstrated the ability of these miRNAs to induce biological responses in target cells.^6,7^ However, their low abundance in EVs makes it less likely that miRNAs account for the majority of functional effects observed.^8,9^ It was later discovered that EV protein cargo, including receptors, transcription factors, or enzymes, could also exert noticeable effects, such as inhibition or activation of downstream pathways in acceptor cells.^10,11^ The transfer of EV-associated proteins may further contribute to the dissemination of the aggressive phenotype among malignant subpopulations within heterogeneous tumors.^12^

EV-associated proteins facilitate intercellular communication and provide valuable insights into the characteristics of their donor cells. EVs encompass an additional dimension—topology—compared with individual proteins alone. The spatial arrangement of EV-associated proteins, either on the surface or in the lumen, offers distinct functionalities in acceptor cells. Normally, EV luminal proteins are internalized into the cytosol of acceptor cells, while EV surface proteins integrate into the plasma membrane of acceptor cells.^13^ Concentrations of EV luminal proteins may reflect treatment efficacy,^14^ whereas EV surface proteins reflect biological responses and EV characteristics. Although analyzing EV proteins in their entirety may reveal their donor cells’ identities (cells-of-origin), “decoding” the spatially compartmentalized proteins (surface versus luminal) of EVs is necessary to fully understand their functionality and potential impacts on acceptor cells. Topology becomes particularly significant for certain classes of proteins. The arrangement of EV surface proteins can inform the transport mechanisms, which may vary under different physiological or pathological conditions of both donor and acceptor cells.^15^ Another potential application of spatial decoding is to examine the presence of EV impurities. Recognizing its significance, spatial decoding has been included as one of the EV characteristics in MISEV2018.^1^

## 2 Application of Tumor EVs

EVs have granted attention for their diverse applications in therapeutics and diagnostics. Here, the focus will be directed toward exploring the potential applications of tumor-derived EVs (“tumor EVs”). As discussed earlier, EVs, with their protective membrane, act akin to “Trojan Horses”, shielding cargo from degradation, driving functional exchange between cells, and extending their molecular content beyond the confines of the cell membrane to both the extracellular space and the cells they reach. Tumor EVs have the potential to activate receptors on tumor-surrounding cells, deliver cargo, and influence these cells to support tumor growth.^16^ While characterizing the protein signatures of tumor EVs can provide a snapshot of the originating tumor cells, existing tools face challenges in dissecting the heterogeneity and scarcity of tumor EVs, as will be discussed in the following section. Notably, given that released EVs exhibit preferences for specific target cells,^17^ tumor EVs may also present tropism toward their parental cells, suggesting a propensity to home back to their origin cells. This property makes tumor EVs an ideal “magic bullet” for targeting the delivery of cancer therapeutics directly to the cancer cells.^18^

During tumorigenesis, tumor cells release a higher quantity of EVs compared to healthy cells. These circulating tumor EVs play a crucial role in tumor metastasis and shaping the tumor microenvironments.^16^ In oncology, traditional liquid biopsies such as circulating tumor cells (CTCs) and circulating tumor DNA (ctDNA) were widely studied. However, EVs offer unique advantages, including long-term stability and storage.^19^ The abundance of tumor EVs in blood far exceeds that of CTCs (<10 CTCs/mL)^21^ and ctDNA concentrations. This abundance positions circulating tumor EVs as attractive “biomarker reservoirs”, providing comprehensive information compared to conventional liquid biopsies and soluble proteins. Moreover, EVs are actively secreted by living cells, whereas ctDNA primarily originates from dead or apoptotic cells.^22^ Examining ctDNA is akin to collecting post-mortem DNA forensics, whereas analyzing tumor EVs is comparable to monitoring active crime activities in real-time. With these advantages, tumor EVs present an enticing option for minimally invasive cancer diagnosis, prognosis, and treatment monitoring across all cancer stages. Their potential as biomarkers for early stage cancer is particularly impactful, as prompt intervention can significantly enhance clinical outcomes.^23−25^ In addition to tumor EVs, neuron-specific EVs represent a compelling option for liquid biopsy in neurodegenerative diseases, offering a viable alternative to the considerable risks linked with brain biopsies in clinical practice.^26^ Analyzing proteins associated with neurodegenerative diseases within neuron-specific EVs isolated from plasma could potentially enable early detection or progression monitoring of diseases such as Parkinson’s Disease and Alzheimer’s Disease.^27^

In addition to diagnosis, tumor EVs can serve as a versatile drug delivery system for cancer treatment.^28^ A distinguishing feature of EV-based drug delivery systems is their capability to overcome physiological barriers in the brain and pancreas, potentially enhancing the therapeutic efficacy of delivered drugs.^29,30^ It is vital to reiterate that one unique feature of tumor EVs is their tumor-targeting properties inherited from parental cell tropism. To date, they have been successfully utilized to deliver drugs deep within tumors.^31−35^ However, progress in engineering tumor EVs for therapeutic purposes or employing them for diagnostics has been hindered by several challenges, including inefficient separation methods, characterization difficulties, and a lack of specific biomarkers, as discussed below.

## 3 Challenges

**Figure 2:**
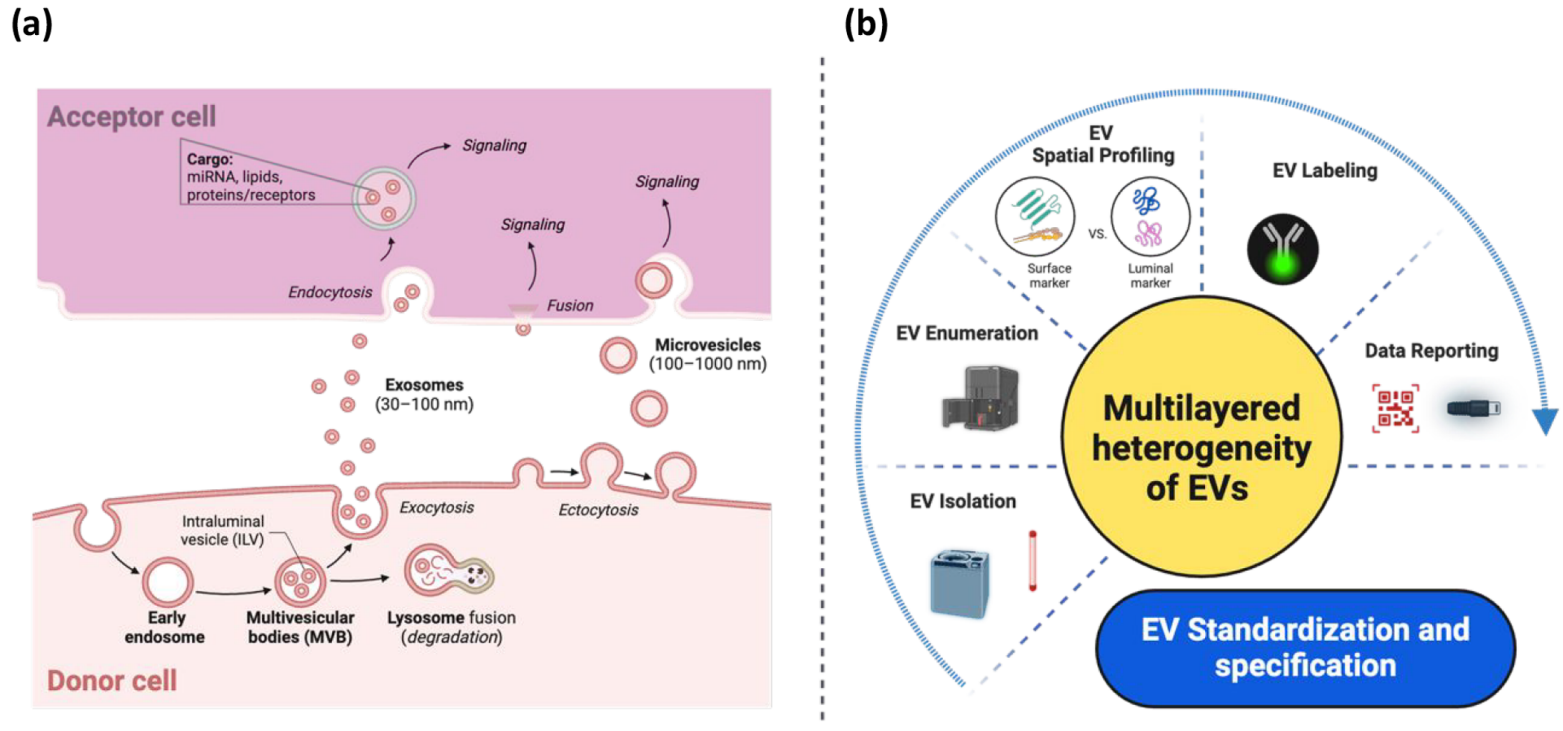
**Challenges encountered in the EV field from both biological and technological perspectives.** (a) The general understanding of the EV-cell interplay, including processes such as EV biogenesis, release, uptake, fusion, and functional transfer between cells. However, detailed mechanisms remain understudied despite this general understanding, which may be attributed to technological limitations highlighted in (b). The collective challenges presented here underscore the complexity of standardizing and specifying EVs for clinical translation.

While EVs offer numerous advantages, limitations in existing methods have left numerous fundamental questions unanswered, which need to be addressed before their widespread use in disease treatment and diagnosis. The key challenges center around the heterogeneity of EVs. In this section, I will highlight these challenges from two orthogonal perspectives: biological and technological (Figure 2).

### 3-1 Biological Perspective: Heterogeneous EV-Cell Interplay

Prior to translating EVs into therapeutics and diagnostics, it is essential to comprehensively understand fundamental EV-cell interactions (Figure 2a).^1^ However, critical aspects of EV biogenesis, release, uptake, fusion, and functional transfer remain understudied, possibly due to technological limitations that will be discussed next. Biological questions to be explored include the dynamics, molecular drivers, and specificity of EV-cell interactions (such as signaling, uptake, or fusion). Knowledge of EV uptake mechanisms may uncover if distinct subsets of EVs utilize different uptake pathways and if key molecules are required in EV trafficking.^25,36,37^ The precise mechanisms through which EV cargo is released into a cell are subjects of current inquiry.^1^ The fate of EV cargoes within acceptor cells remains largely unexplored, including whether they bypass lysosomal degradation and are released into the cytosol or are recycled within newly formed EVs for resecretion into the extracellular media (Figure 2a). The field should be cautious about being overly focused on fusion, potentially neglecting lysosomal degradation or signaling pathways. This could create a misleading impression regarding the primary fate of EVs and the significance of cytosolic cargo delivery.

The effects of EVs on acceptor cells typically begin with EVs binding to receptors either on the cell surface or by releasing their cargo into the cell (Figure 2a). Membrane-associated proteins play a crucial role in facilitating the uptake of EVs into cellular compartments.^38,39^ However, the uptake of EVs by most nonphagocytic cells may occur at a low rate,^40,41^ necessitating a high ratio of EVs to acceptor cells to observe the EV-cell interplay.^42^ Additionally, factors such as dose, time, pH, and temperature influence the uptake of EVs by cells. EV uptake can occur rapidly, with a time frame as short as 15 min, but is typically observed over a 4-h period to align with endocytosis rates.^43^ It is important to note that using a fixed EV dose for different acceptor cell types may yield misleading results. Introducing high concentrations of proteins or RNAs into cell lysates may lead to physiologically irrelevant associations or induce off-target effects. Establishing reproducible in vitro systems that closely mimic the physiological context is essential for studying EV cargo sorting mechanisms, which remains unclear.^44,45^

There are layers of biological heterogeneity to be considered in EV research. These include variations in parent cell clones and cell types, vesicle size, EV biogenesis pathways, EV shedding kinetics, and molecular content packaged into individual EVs. Each EV carries a unique composition and distinct information set, much like individual cells. While these EVs can be categorized based on shared features, such as size, surface molecules, internal cargo, cell of origin, or function, variability exists among vesicles within each category. Despite the potential of specific lipids and membrane proteins to indicate their origin,^46,47^ it remains uncertain if phenotypic heterogeneity corresponds to composition variability. This poses challenges in evaluating their physiological relevance, considering factors like origin, biogenesis, and secretion mechanisms. Unlike classical information transfer in physiology, where identical molecules (e.g., hormones) generate signals in sufficient concentrations, EVs follow a more complex pattern. They enter cells through endocytosis or phagocytosis, where they may be degraded in lysosomes or resecreted (Figure 2a). Once secreted, these EVs contribute to the overall circulating EV pool. Viewing challenges as opportunities, the heterogeneity of EVs and their cargo signifies the “enhanced potential” for ushering in a new era of information exchange between cells. These unique protein-decorated phospholipid vesicles, compared with other lipid-bound nanoparticles, contain specific barcodes necessary to locate their targets locally and at distant sites, enabling multifunctionality.^48^

### 3-2 Technological: Limitations and Strategies for Studying EVs

The field of EV research is still in its infancy, with robust tools for studying these inherently heterogeneous and nanosized vesicles steadily advancing.^49^ Despite this progress, no universal markers have been identified to precisely define ectosomes, exosomes, or other EV subtypes.^1^ The lack of consistent reporting and terminology for heterogeneous EVs has resulted in uncertainties and controversies in the technologies and experimental designs in the EV field. Below are some common technological limitations encountered when studying EVs, along with strategies to address them (Figure 2b).^1,50−53^

After collecting EVs, these vesicles can be isolated (or termed “concentrated,” “enriched,” or “purified”) according to their biophysical characteristics, including size, density, charge, and surface composition.^1^ However, currently available isolation techniques may not yield entirely pure EV samples, as there is a significant potential for coisolating impurities, especially various classes of lipoproteins. Different EV isolation protocols encounter unique challenges and may produce varying purity levels in isolated EV samples, leading to discrepancies in reported EV concentrations. For instance, size-exclusion chromatography (SEC) coelutes large lipoproteins with EVs due to their overlapping size distributions.^20^ The polyethylene glycol (PEG) precipitation method may also coprecipitate apoB-containing particles such as very-low-density lipoproteins (VLDL) and low-density lipoproteins (LDL) with the EVs, yielding higher EV concentrations than values obtained by ultracentrifugation.^20,50,54^ Even ultracentrifugation, a standard method for EV isolation, is not exempt from this issue and may introduce other artifacts, such as disruption of membrane topology and EV aggregation.^55^ Lipoproteins are unignorable EV impurities due to their physical properties overlapping with EVs and their significantly higher concentration in plasma than EVs.^56^ The total amount of lipoproteins in human plasma is around 10^16^ particles per mL, which is about 6 orders of magnitude higher than the EV concentration in plasma and significantly exceeds the count of rare tumor EVs.^20,57^ Even techniques like nanoparticle tracking analysis (NTA), which is considered the gold standard for determining EV concentrations and size distributions,^1,50,54^ struggle to distinguish between EVs and lipoprotein contamination. Therefore, when measuring EV concentrations in plasma, significant variations can be easily introduced due to the low abundance of EVs in these environments, contributing to variations in reported EV concentrations and complicating comparisons between studies.

Unlike most biomolecules, there is no universally accepted baseline value for EV concentration, further complicating data interpretation.^53,58,59^ Reported EV concentrations in the blood of healthy individuals can vary widely from 10^8^ to 10^13^ EVs per mL (with an average of approximately 10^10^ EVs per mL).^20^ To address these challenges, researchers may employ multistep isolation protocols based on various physical parameters to increase EV purity.^57,60^ However, such stringent protocols are like double-edged swords that run the risk of overpurifying EVs or selectively isolating more stable EVs.^44^ This overpurification could inadvertently remove important signaling molecules or cofactors, affecting downstream studies. There’s not yet a universal consensus on best practices for EV counting and sizing. Beyond NTA, other EV counting and sizing methods encompass tunable resistive pulse sensing (TRPS), flow cytometry, single-particle interferometric reflectance imaging sensing (SP-IRIS), and electron microscopy. Each method has its own strengths and weaknesses, with biases toward specific EV size ranges. Note that size alone is insufficient for the definitive categorization of EV subpopulations.^1^ A comprehensive approach considering multiple physical parameters beyond size is necessary for thorough EV characterization.

Proteins constitute important cargo carried by EVs. Ongoing multiomics-based approaches may facilitate the discovery of cell-specific markers, essential for tracing EVs back to their cells of origin. However, progress is often impeded by emerging challenges, leading to setbacks. Uncertainties persist regarding specific markers for distinct EV subpopulations across cell types. While specific proteins like TyA, C1q, and CD73 have been proposed as potential markers for EVs derived from cell membranes, and tetraspanins (CD61, CD63, CD81) for EVs originating from endosomes, the presence of endosomal proteins traversing the cell membrane complicates the identification of EV subpopulations. Moreover, soluble forms of these EV-associated proteins in the blood,^47,61,62^ along with potential nonspecific binding of reagents and intrinsic noise in complex clinical samples, pose challenges for studying cell-specific EV markers. These factors introduce background signals and raise questions about the true extent to which a given protein is contained within EVs versus being present outside of them.^63^ In addition, RNA and DNA have been reported to associate with the outside of the EV membrane, potentially due to artifacts of the isolation procedure.^51^ A classic example is the L1CAM protein, previously considered a common neuronal EV marker for immune-isolation,^26,64^ but later found not to be associated with cerebrospinal fluid and plasma EVs by the Walt group.^65^

One distinguishing feature of EV-associated proteins is their sublocalization within discrete spatial compartments, leading to another layer of biomarker. These proteins can reside on the outer surface of EVs, be attached to the inner membrane layer, or be enclosed within the EV lumen.^13^ This spatial distribution of EV-associated proteins influences their biochemical properties and roles in cancer progression.^66^ For instance, luminal proteins may include mutant tumor suppressor proteins, oncoproteins, and critical signal transduction mediators, suggesting their potential as highly specific cancer biomarkers.^67^ Techniques such as protease and nuclease digestions, detergent permeabilization, and antibodies targeting outer or inner epitopes can probe their compartments separately. Additionally, mass spectrometry can theoretically detect EV luminal proteins. However, quantifying these EV-associated proteins in blood samples is challenging due to the significantly higher levels of other plasma proteins (e.g., albumin and immunoglobulins). Unique strategies are needed to accurately characterize these proteins based on their sublocalization within EVs. It is recommended to combine techniques such as Western blot, electron microscopy, live imaging, and pH sensors to identify specific protein markers of distinct EV subtypes released from or even within specific cell types.

As EVs typically exhibit a higher protein-to-lipid ratio than their donor cells, they are often reported using weight-based units (μg EV protein) or weight concentration (μg/mL) rather than molar concentration. However, this indirect quantitative approach complicates the correlation between in vitro and in vivo situations, where molar concentration would be more straightforward for understanding physiological functions. For instance, based on estimations from our laboratory data on EVs released by various pancreatic cancer cell lines, 1 μg EV protein corresponds to approximately 10^9^ EVs. Applying these estimates to evaluate data from a typical EV study, 1000 μg EVs (equivalent to 10^12^ EVs) were added to about 10^6^ cells, resulting in about 10^6^ EV particles per cell. This raises concerns regarding high EV concentrations and EV per cell ratios in real organisms. So, why do these EV studies use weight-based units instead of moles? This choice may stem from the technological limitations of existing analysis methods, primarily the limited sensitivity and specificity. For example, as discussed earlier, NTA may not always be ideal for quantifying EV concentrations. In such cases, a bicinchoninic acid (BCA) protein assay may be an alternative to quantify EV proteins as an indirect quantification index. However, the limited sensitivity of this method could pose another challenge: high EV concentrations are often required in experiments to meet the sensitivity threshold, potentially leading to biologically irrelevant outcomes in these studies.

In cancer, tumor EVs constitute only a minute fraction of the total pool of circulating EVs released by various cells (although a tumor cell releases more EVs than a healthy cell). Identifying these tumor EVs among the others is like finding “a needle in a haystack”. Furthermore, only a subset of established cancer markers is present in circulating tumor EVs, and their abundance is often very low, particularly in early stage cancer.^68^ Therefore, it is difficult to discretely map the secretion of rare tumor EV subpopulations to their parent cell of origin. These challenges increase dramatically in the intratumoral microenvironment, where distinct cell subpopulations exhibit diverse biological profiles. Due to the limited sensitivity of existing methods, extensive preprocessing steps are often needed, such as ultracentrifugation, density gradient centrifugation, and SEC, to enrich tumor EVs prior to subsequent characterization. However, this workflow is time-consuming, labor-intensive, and lacks precision, posing challenges for translation to a large scale in clinical settings. Moreover, these methods typically analyze tumor EVs as a bulk population and cannot dissect the inherent heterogeneity among tumor EV subpopulations.

In vivo EV studies offer valuable mechanistic insights into EV release, biodistribution, pharmacokinetics, and function. However, tracing EVs in vivo is often challenging due to the lack of efficient pan-EV markers. One common approach involves labeling EV proteins, such as tetraspanins, with fluorescent proteins like GFP. However, given the estimated low abundance of specific proteins on the EV surface, with fewer than 100 copies depending on EV size, labeling with fluorescent proteins can be challenging and may lead to potential false negatives.^69^ Alternatively, fluorescent lipid dyes such as PKH or Bodipy can be used to label the lipid membrane of EVs.^70^ When employing this labeling approaches, several factors must be considered, including the half-life and brightness of the fluorescent tag, the resolution limits of the microscopy technique, potential quenching effects due to the small surface area of EVs, and the possibility of altering EV cargo or function through labeling. Additionally, general EV membrane labeling may inadvertently label non-EV components like lipoproteins or cell debris, leading to false positives. As there is no universal labeling strategy for EVs, protocols for optimal EV labeling practices are needed to discern between labeled EVs, dye aggregates, and contaminants such as those found in BSA.^71^ Including free dye controls in such experiments is crucial to demonstrate the clearance of unbound dye and ensure that all observed fluorescent entities are associated with EVs.

The inherent multilayered heterogeneity of EVs poses significant challenges in establishing a gold standard for their standardization.^50,54^ The molecular contents of EVs can vary significantly based on their organ source and cell of origin. EVs can exhibit heterogeneity even when originating from homogeneous or monoclonal cell populations.^72^ Furthermore, the temporal expression of analytes within a defined EV population can vary considerably.^68^ Identifying tumor EVs in early stage cancers is difficult due to their scarcity compared to EVs shed from a pool of healthy cells. Additionally, nanoscale particulates such as protein aggregates and cell debris in clinical samples can overlap with EVs, leading to potential false positive results. In addition to these challenges related to heterogeneity and specificity, the limited capacity of EVs to package molecular cargo requires the analysis of large numbers of EVs for downstream studies. Overall, this absence of a standardization protocol complicates EV manufacturing in the pharmaceutical industry, particularly when transitioning from small laboratory-scale batches to large industrial quantities for the development of EV-based therapeutics or diagnostics. Each step introduces complexities. For example, the conditions under which cells are cultured can significantly influence the characteristics of the released EVs. For EV-based therapeutics to be translated into clinical applications, they must comply with regulatory standards and adhere to current good manufacturing practices (cGMP). Maintaining the quality and consistency of EVs on an industrial scale necessitates further optimization of production processes, from cell culture to EV isolation and purification.

## 4 Strategies for Translating EVs into Personalized Diagnostics and Therapeutics

Recognizing the multilayered heterogeneity of EVs as both limitations and opportunities of EVs, a new focus is being placed on translating them into personalized diagnostics and therapeutics. Bulk EV analysis, which provides an average behavior as seen with most current methods, falls short in resolving the heterogeneity of individual EV subpopulations, especially rare ones, from clinical samples. Hence, new technologies are delving into analyzing single EVs. This shift is crucial for realizing personalized medicine, as each EV carries unique characteristics, and only by examining them individually can we discern variations in their frequencies, sizes, or protein content. However, analyzing single EVs is challenging due to their small size (below the diffraction limit of light) and limited molecular content. While advanced super-resolution microscopy methods have emerged to overcome these challenges,^68,73^ they still have limitations, including extensive EV labeling and purification procedures and limited sensitivity. Therefore, to address these technical gaps, single-EV or “digital” EV profiling methods^74−88^ have been developed, offering improved sensitivity, throughput, multiplexing capacities, and requiring minimal prepurification steps. For example, Lin et al. developed a dual-target aptamer detection probe specific for EpCAM and PD-L1, enabling a quantitative proximity ligation assay (PLA) to assess PD-L1 expression on tumor-derived EVs.^83^ He et al. introduced an ultrasensitive single EV assay that can visualize and quantify tumor EVs directly from plasma using activatable aptamer probes triggering fluorescence.^87^ Wei et al. utilized Simoa to purify tumor-derived EVs (EpCAM-CD63), demonstrating superior diagnostic performance for colorectal cancer compared to traditional serological biomarkers like CEA and CA125.^88^ We foresee that these digital EV profiling methods could offer insights into unsolved fundamental biological questions, such as EV transfer and cargo delivery mechanisms, both in vitro and in vivo. Moreover, for rare tumor EV subpopulations lacking specific cancer markers, which makes tracing back to the cell of origin challenging, digital EV profiling methods can be combined with single-cell approaches to identify diverse EV subsets derived from single-cell clones.

To enhance the therapeutic potential of EVs, efforts are underway to improve their cargo loading capacity, circulation time, and targeting capability, aiming to reduce the number of EVs required to achieve ideal therapeutic efficacy. Cargo loading into EVs can occur intracellularly (top-down) or extracellularly (bottom-up). In the former approach, donor cells are manipulated to load the cargo, while in the latter, cargo is added to the isolated EVs through experimental interventions like electroporation and surfactant- or pH-dependent opening of EV membrane. The choice of loading approach can significantly impact the subsequent efficacy of the therapeutic cargo in acceptor cells, with each approach being ideal for specific classes of therapeutic cargo. For example, hydrophobic drugs like erlotinib may be more feasible to add directly to EVs rather than loading them through cellular manipulation. In vivo, exogenously administered EVs exhibit a relatively short half-life in circulation, typically lasting only tens of minutes and often necessitating serial dosing.^44^ Strategies such as PEGylation or incorporating a “don’t eat me” signal, such as CD47,^30^ are commonly employed to increase the half-life of EVs.

Unlike cells, EVs cannot actively seek targets via signal gradients, so the concept of “EV targeting” should be described cautiously to avoid misleading. Upon entering the human body, EV distribution is mainly driven by passive accumulation or passive targeting, determined by the different affinities for cells encountered by chance. EVs can trigger immune responses and may be engulfed by the reticuloendothelial system, leading to selective enrichment in tissues such as liver, spleen, lung, and bone marrow. In certain cancers, injected EVs may accumulate in inflamed tissue due to vascular leakiness.^30^ EVs are more likely to enter the brain during inflammation.^89^ Their accumulation within kidneys significantly increases in animal models of acute kidney injury. It is worth noting that plants can also produce EVs apart from humans and animals. These plant-derived EVs share a similar structure with those isolated from mammals and can transport various molecules such as mRNAs, miRNAs, bioactive lipids, and proteins to animal cells.^90^ One significant difference between mammalian-derived and plant-derived EVs is that the latter are considered safe due to being natural nanoparticles secreted by plants and already present in foods. Moreover, plant-derived EVs carry cargo less related to human EVs, potentially resulting in a lower chance of triggering physiological or pathophysiological responses, such as immune responses.^91^ Therefore, plant-derived EVs could also be engineered for drug delivery applications.^92^

**Figure 3:**
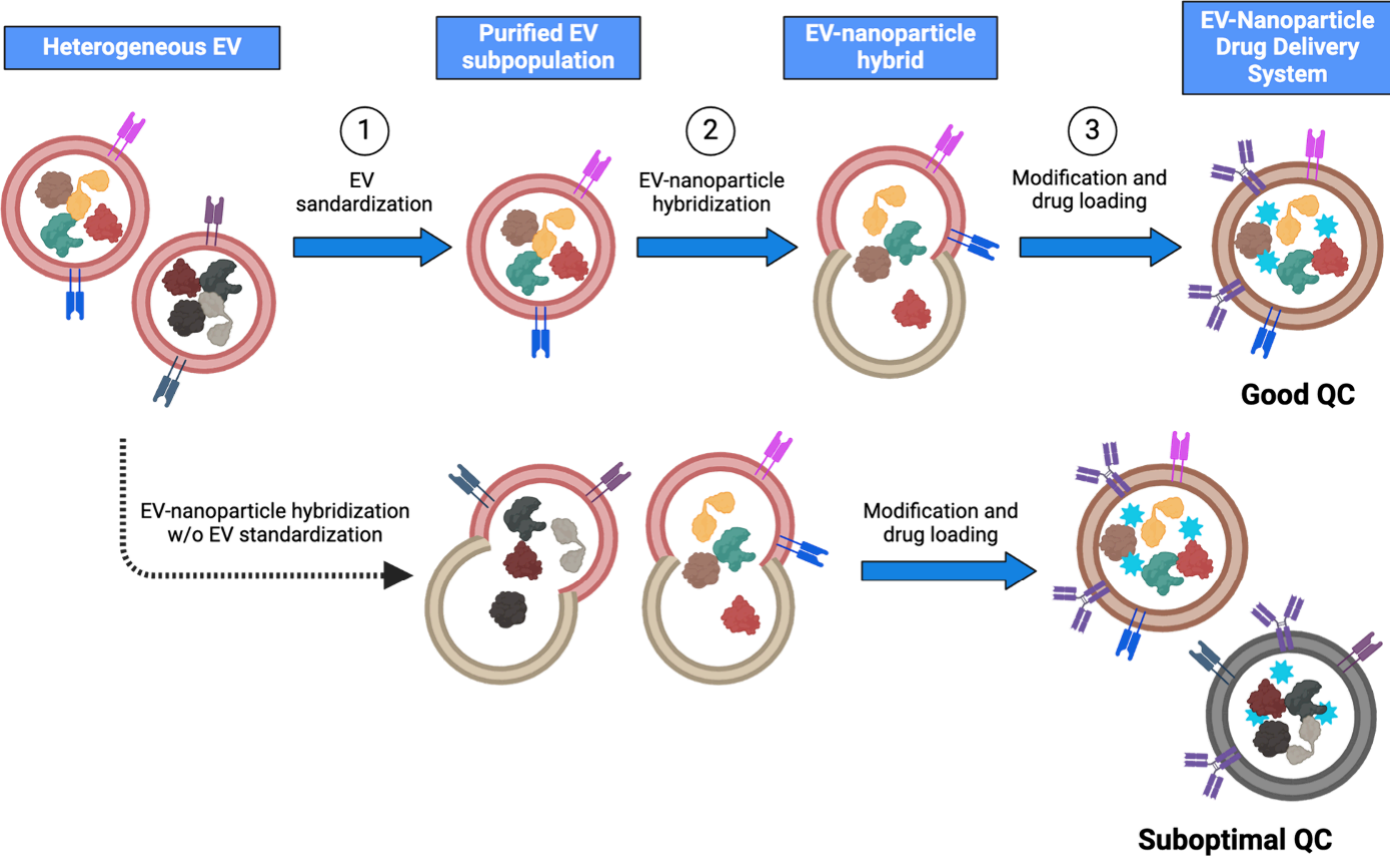
**Potential engineering workflow of EV-nanoparticle drug delivery system for personalized therapeutics. Step 1 (top):** Heterogeneous EVs released from donor cells undergo EV standardization to purify and recover the EV subpopulation of interest. **Step 2:** The recovered EV subpopulation with improved uniformity will be hybridized with nanoparticles to form an EV-nanoparticle hybrid. **Step 3:** To enhance the targeting ability of the hybrid particle, ligands will be modified to the particle’s surface, and the drug will be loaded into the EV-nanoparticle hybrid. This resulting EV-nanoparticle hybrid exhibits improved uniformity and thus better quality control compared to the hybrid lacking EV standardization (bottom).

Novel strategies have emerged for developing EV hybrid vesicles to further improve drug loading capacity and introduce multifunctionality. One common approach involves fusing natural EVs with artificial nanoparticles (like, liposomes), resulting in EV-nanoparticle hybrids (Figure 3). With nanoparticles’ known physical and chemical properties, these hybrids are expected to be manageable in synthesis and characterization. They offer expanded cargo space to accommodate larger payloads such as CRISPR/Cas9 expression vectors and high-molecular-weight drugs. They are preferred over EVs alone when delivering hydrophobic drugs, as specific nanomaterials can provide hydrophobic domains suitable for loading such drugs, complementing the hydrophilic central core of EVs. Combining the biomimetic nature of EVs with the versatility of artificial nanomaterials, these EV-nanoparticle hybrids possess pleasing properties, including prolonged circulation time, enhanced permeability and retention (EPR) effect, modifiability, and intelligent responsiveness to stimuli such as heat, magnetic, or ultrasound. Moreover, they enable the codelivery of multiple drugs, enhancing efficacy while minimizing toxicity and side effects, particularly when coupled with targeting molecules to achieve site-specific drug delivery. Similarly, modifying EV-nanoparticle hybrids with specific ligands can reduce their uptake and clearance by immune cells. These hybrids can serve as a theranostics platform by incorporating luminescence, ultrasonic signaling, and magnetic properties. The application of EV hybrid vesicles for therapy and diagnosis is still in its early stages, requiring further research to optimize their in vivo performance. It is crucial to consider safety, biocompatibility, and biodegradability carefully to prevent the accumulation of artificial nanomaterials in the body. To achieve improved uniformity and enhance quality control, EV standardization can play a crucial role in purifying and recovering the EV subpopulation of interest from the heterogeneous bulk of EVs (Figure 3). This process ensures that the EVs used for hybridization with nanoparticles exhibit enhanced uniformity and quality before forming the EV-nanoparticle hybrids. Once the synthesis and characterization processes are standardized, EV hybrid vesicles are anticipated to become a significant focus of EV-based therapeutics in the coming future.

**Figure 4:**
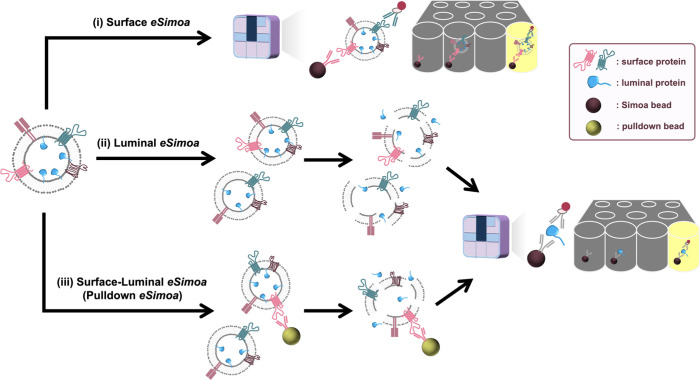
**Workflow of the EV single-molecule array (eSimoa) framework for spatial decoding of EV-associated proteins.** The eSimoa framework combines EV isolation with ultrasensitive protein detection to profile EV proteins with exceptional sensitivity and specificity. The eSimoa framework comprises three complementary pipelines. Pipeline (i): surface eSimoa, capturing and detecting two EV surface proteins. Pipeline (ii): luminal eSimoa, analyzing EV luminal proteins. Pipeline (iii): surface-luminal eSimoa or pulldown eSimoa, integrating the surface and luminal eSimoa approaches by selectively targeting a subpopulation of EVs with a specific surface protein using pulldown beads, followed by the analysis of luminal proteins within this subpopulation. Reproduced from ref (77). Copyright 2023 The Authors, published by Wiley-VCH GmbH under a CC-BY 4.0 license.

Proteins located on different regions of EVs (surface versus luminal) exhibit unique functionalities, contributing to the distinctiveness of individual EVs. “Spatially decoding” these proteins may provide a more accurate reflection of the cell-of-origin for a specific EV and its potential effects on acceptor cells. To advance EVs’ diagnostic and therapeutic potential in cancer research, our team has pioneered innovative digital EV profiling approaches to decode these EV protein biomarkers at single EV resolution, collectively known as the eSimoa framework (Figure 4).^77^ This framework seamlessly integrates EV isolation with ultrasensitive single-molecule array (Simoa) protein detection technology.^93^ Simoa, one of the digital ELISA methods, is renowned for its unrivaled sensitivity, capable of quantifying proteins at attomolar concentrations, representing a million-fold improvement over existing methods.^93^ The significance of this work lies in its potential to advance the field of EV research toward personalized diagnostics and therapeutics, highlighted as a Front Cover Story in *Advanced Science*.^77^ The eSimoa framework (Figure 4) consists of three complementary and orthogonal pipelines: surface eSimoa, luminal eSimoa, and surface-luminal eSimoa (pulldown eSimoa). The “surface eSimoa” pipeline captures and detects EVs based on two surface protein biomarkers, ensuring that only EVs harboring both surface proteins undergo downstream analysis. The “luminal eSimoa” pipeline analyzes EV luminal proteins, while the “surface-luminal eSimoa” pipeline captures EVs with specific surface proteins and analyzes the luminal proteins of this targeted EV subset. Together, these three pipelines enable the profiling and quantification of surface and luminal EV proteins, providing a comprehensive understanding of their spatial distribution within EVs. Utilizing this framework, we have detected EVs at concentrations as low as 10^5^ EV/mL in plasma while quantifying absolute EV protein concentrations as low as fM. This study targeted CD81 and CD63 as surface proteins, and RAS and KRAS^G12D^ as luminal proteins within EVs. We successfully identified KRAS^G12D^ as a potential EV protein biomarker for pancreatic cancer. The exceptional sensitivity and versatility of the eSimoa framework offer unprecedented opportunities to discover and validate novel EV protein biomarkers. Furthermore, its direct applicability to clinical samples with minimal prepurification steps paves the way for developing minimally invasive blood tests for various diseases, including cancer. One of the most unique features of eSimoa is its ability to provide ABSOLUTE quantification of rare EV proteins in plasma, differing from the semiquantification obtained by other methods such as mass spectroscopy and western blot. This absolute quantification is especially important for EV standardization and specification, prerequisites for translating EVs into diagnostics and therapeutics.

Now, returning to the concept of the EV Trojan Horse; while it is widely accepted that EVs facilitate intercellular communication by transporting cargo (Figure 2a), the specific mechanisms involved in EV delivery within acceptor cells and the subsequent release of biomolecules remain lacking. Addressing these gaps is essential, given the high translational impact of EVs. We anticipate that innovative strategies, such as an EV multiplexed protein analysis platform, could offer high-resolution protein signatures of EVs. This advancement would greatly facilitate our understanding of the intricate interplay between EVs and cells. Particularly, EVs released by tumor cells harbor distinct “protein signatures”, including oncoproteins, compared to those from other cells. It is crucial to study whether these oncoproteins may also be transferred via the EV Trojan Horse mechanism and affect downstream signaling and phenotypes in acceptor cells.

Multiplexed assays have the potential to significantly contribute to EV standardization by providing multilayer profiles of EV signatures. However, it is essential to note that most current multiplexed assays^68,73^ were designed for diagnostic purposes and did not recover the well-characterized EVs. This means that these valuable EVs are often wasted without further utilization. Therefore, future efforts should prioritize streamlining EV standardization and the subsequent recovery processes. The well-standardized EVs could then be employed as EV therapeutics, as shown in Figure 3. Success in this endeavor would greatly benefit the development of EV-based therapeutics and, perhaps, diagnostics.

## 5 Perspectives and Conclusions

**Figure 5:**
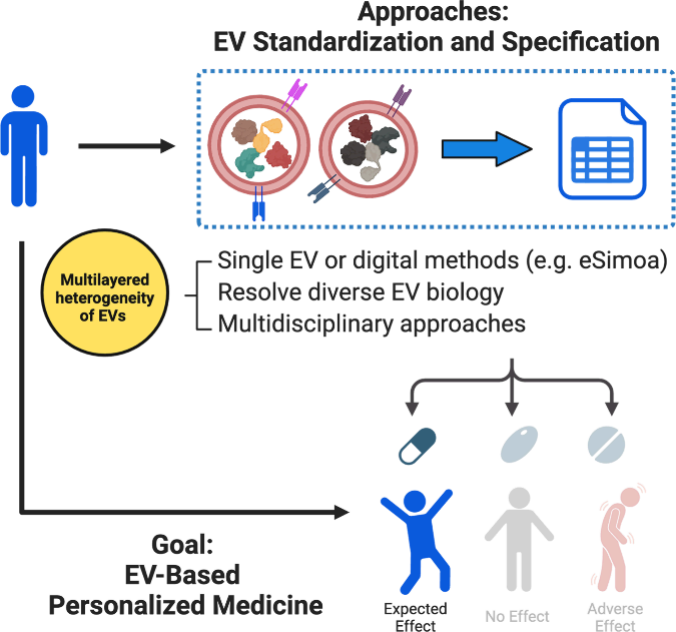
**Proposed strategies to advance EV-based personalized medicine.** Because of the multilayered heterogeneity of EVs, thorough EV standardization and specification are essential prerequisites for translating EVs into therapeutics. This can be achieved from three perspectives: leveraging advanced technologies such as single EV or digital methods (e.g., eSimoa), deepening our knowledge of diverse EV biology, and embracing multidisciplinary approaches. Once fulfilled, such EV therapeutics has the potential to become next-generation personalized medicine.

The urgency for personalized medicine is increasingly evident, especially in complex diseases like pancreatic cancer, where patients with distinct mutations often exhibit varied responses to treatments. In addressing this critical need, the potential of EV-based personalized therapeutics and diagnostics is emerging as a promising avenue. Before proceeding with clinical implementation, thorough EV standardization and specification are essential (Figure 5). Current EV studies typically involve isolating and purifying EVs before characterizing them in a separate technology platform. However, no methodology for EV isolation and purification achieves ideal yield, purity, ease of use, and scalability simultaneously. This dilemma creates significant trade-offs in existing EV isolation approaches, posing a bottleneck in the clinical application of EVs. We must acknowledge the reality that isolating pure or homogeneous vesicle populations is challenging due to the inherent heterogeneity of EVs.^94,95^ Recognizing EVs and lipoproteins as part of a continuum of lipid-containing particles and are difficult to separate is also essential, especially when relying on the concentrations of EVs or their cargo in plasma as biomarkers.^56,57^ A protein contaminant repository for affinity purification may be a good reference to look up for non-EV interferants.^96^ Future research efforts should prioritize optimizing isolation protocols to obtain purer EV samples, thereby enhancing correlations with disease states and bolstering clinical specificity, especially in cancer. New protocols that leverage the multifaceted physicochemical characteristics of EVs could improve sample purity and enhance the quality of EV biomarkers. Yet, multistep methods may lead to a loss of information fidelity.

One of the “holy grails” in EV research is to achieve ultrasensitive detection of EVs directly in clinical samples without the need for complicated sample preprocessing. Automation technologies that streamline EV isolation and ultrasensitive EV analysis into a single platform, such as our eSimoa framework, hold promise for enhancing reproducibility and diagnostics performance. These advancements could lead to more efficient analyses with smaller sample sizes and reduced assay times. Our eSimoa framework (Figure 4) not only provides a novel tool for studying cancer-specific EV protein biomarkers in clinical samples with minimal prepurification steps,^77^ but also enables comprehensive profiling and quantification of surface and luminal EV proteins, providing a detailed landscape of their spatial distribution within tumor EVs. The application of Simoa technology in EV-related cancer research is still nascent, with only seven previous studies identified.^88,97−102^ However, with its “best-in-class” ultrasensitivity and ability to greatly simplify the EV research workflow, the eSimoa framework stands out as a promising tool in the emerging EV field. Notable applications of eSimoa include, but are not limited to, (i) characterizing EV subpopulations, (ii) profiling and quantifying EV cargo with absolute precision, and (iii) establishing EV standardization and specification for quality control purposes, such as assessing EV yield and purity. These unique capabilities of eSimoa could propel EVs as next-generation liquid biopsies for diagnostics and provide EV biomaterials with enhanced uniformity and quality for EV-based therapeutics. As a result, eSioma serves as a powerful platform for translating EVs into personalized diagnostics and therapeutics.

Leveraging the combined capabilities of eSimoa^77^ and nanoSimoa,^103^ two complementary tools recently developed by our group, we can unravel the intricate interplay between EVs and cells at the molecular level. By elucidating personalized EV signatures and EV interactions with cells, we can tailor EV-based drug delivery platforms to individual patients, thus advancing precision medicine. For instance, unique EV protein signatures for each individual can aid in patient stratification, facilitating the selection of personalized treatment plans based on individual’s needs and characteristics (Figure 5). To address the multilayered biological heterogeneity among EV subpopulations, advancing “digital” assays or single EV profiling technology should be our next focus. This optimization involves balancing sensitivity, throughput, and multiplexing capabilities. One potential opportunity lies in developing an integrated and continuous “lab-on-a-chip” workflow that combines EV enrichment, signal amplification, and signal detection into a single pipeline. Machine learning algorithms can aid in interpreting large volumes of data generated from a single EV analysis and reduce discrepancies among methods.

Embracing a multidisciplinary approach is another key strategy for advancing EV research (Figure 5). By bridging experts from diverse disciplines such as chemistry, pharmaceutical science, medicine, and bioengineering, we can unlock valuable insights and drive innovation. Our research group, for example, comprises researchers from these varied backgrounds, recognizing the immense potential of collaborative efforts in pushing the boundaries of EV research. One particularly promising area for interdisciplinary collaboration lies in leveraging insights from virology. Collaborating with virologists can deepen our understanding of EV release and uptake pathways, as there are striking similarities between the assembly and disassembly processes of enveloped viruses and EV dynamics. Drawing parallels with virology, we can potentially inform the design of artificial EVs tailored for specific cells and develop assays to monitor the delivery of RNA or protein into the cytosol.

Finally, standardized protocols, quantification, and transparent reporting are crucial to ensuring meaningful and reliable EV studies that can be translated into clinical applications. As cell-based therapies have received regulatory approval, we anticipate similar support for EV therapeutics from regulatory agencies. Before translating into clinical applications, transparent reporting methods should be conducted to reconcile conflicting data across different laboratories. To achieve this, it is essential to adhere to the recommended reporting guidelines outlined in the MISEV by ISEV.^1,50,54^ These guidelines provide a framework for comprehensive reporting of details in each EV study, facilitating transparency and enabling critical evaluation of consistency across experiments. The EV-TRACK consortium has also established a knowledgebase and coaching tool to promote transparency and reproducibility in EV methods.^53^ By implementing thorough specification and detailed reporting practices, the field can enhance replicability and reproducibility, ultimately embracing the robustness and reliability of EV research. Our eSimoa framework, designed to enable absolute quantification of EV proteins across a wide range of abundances, will benefit the standardization and specification of EVs. Through continued adherence to rigorous reporting standards and the adoption of innovative methodologies like eSimoa, the EV community can propel the translation of EV-based therapeutics and diagnostics into clinical practice with confidence and clarity.
